# Enhanced Functional Properties of Bioplastic Films Using Lignin Nanoparticles from Oil Palm-Processing Residue

**DOI:** 10.3390/polym14235126

**Published:** 2022-11-25

**Authors:** Samsul Rizal, Tata Alfatah, H. P. S. Abdul Khalil, Esam Bashir Yahya, C. K. Abdullah, Eka Marya Mistar, Ikramullah Ikramullah, Rudi Kurniawan, R. D. Bairwan

**Affiliations:** 1 Department of Mechanical Engineering, Universitas Syiah Kuala, Banda Aceh 23111, Indonesia; 2 Bioresource Technology Division, School of Industrial Technology, Universiti Sains Malaysia, Penang 11800, Malaysia; 3 Green Biopolymer, Coatings & Packaging Cluster, School of Industrial Technology, Universiti Sains Malaysia, Penang 11800, Malaysia; 4 Bioprocess Technology Division, School of Industrial Technology, Universiti Sains Malaysia, Penang 11800, Malaysia

**Keywords:** bioplastic, hydrophobicity, antimicrobial, lignin nanoparticles, purification, *Kappaphycus alvarezii*, packaging

## Abstract

The development of bioplastic materials that are biobased and/or degradable is commonly presented as an alleviating alternative, offering sustainable and eco-friendly properties over conventional petroleum-derived plastics. However, the hydrophobicity, water barrier, and antimicrobial properties of bioplastics have hindered their utilization in packaging applications. In this study, lignin nanoparticles (LNPs) with a purification process were used in different loadings as enhancements in a *Kappaphycus alvarezii* matrix to reduce the hydrophilic nature and improve antibacterial properties of the matrix and compared with unpurified LNPs. The influence of the incorporation of LNPs on functional properties of bioplastic films, such as morphology, surface roughness, structure, hydrophobicity, water barrier, antimicrobial, and biodegradability, was studied and found to be remarkably enhanced. Bioplastic film containing 5% purified LNPs showed the optimum enhancement in almost all of the ultimate performances. The enhancement is related to strong interfacial interaction between the LNPs and matrix, resulting in high compatibility of films. Bioplastic films could have additional advantages and provide breakthroughs in packaging materials for a wide range of applications.

## 1. Introduction

To date, plastics have become the widely used products of choice due to their high levels of stability, affordability, and functionality. However, the consumption of synthetic plastic materials derived from raw petrochemicals has a severe impact on environmental pollution since the majority of synthetic plastics are not easily degraded by microbial decomposers [1]. Although synthetic plastics can physically be broken down into smaller particles in environmental media such as landfills, soil, water, and marine environments, they accumulate in the food-chain system, causing various diseases. Recently, numerous approaches have been devoted to developing the synthesis of biodegradable polymers. One such approach is bioplastics, a rapidly growing class of polymeric compounds that are produced from renewable feedstock and are both environmentally sustainable and functionally similar to synthetic plastics [2]. Therefore, bioplastics derived from renewable sources have been developed to alleviate the synthetic plastic pollution problem, as the majority of bioplastics can degrade whenever exposed to bioactive environments such as compost and soil [3]. Recent studies reported on biodegradable plastic films fabricated from biomass feedstocks, such as chitosan [4], alginate [5,6], starch [7], and seaweed [8]. Such prior publications revealed a considerable improvement in the bioplastic films’ properties.

Biopolymers are natural polymers derived from plants and animals, which include a variety of polysaccharides, polypeptides, and polynucleotides [9], while biomonomers are small molecules (i.e., monomeric subunits) that can undergo ex situ chemocatalytic polymerization to produce biobased polymers [10]. The utilization of biopolymers is not limited to bioplastics but ranges from sustainable production of other materials such as biofuels, bio-implants, and medicinal products [11]. Seaweed is considered one of the most potential biopolymers. *Kappaphycus alvarezii*, a red seaweed, is an eco-friendly, abundant, sustainable, and low-cost bioresource. The chemical composition of *Kappaphycus alvarezii* was 65.20% carbohydrate, 11.57% ash, 3.40% protein, and 1.10% lipid contents [12]. In this sense, carrageenan is a natural carbohydrate (polysaccharide) and commercially essential phycocolloid generated from red seaweeds. The extraction and purification process of carrageenan is expensive, time-consuming, and requires abundant chemicals and water during processing [13]. To overcome these issues, raw red seaweed was used for the development of bioplastic films since it is cheaper and easier to process than pure carrageenan.

Recent studies have reported that raw seaweed can actually form a bioplastic film with adequate ultimate performance [12,14]. However, bioplastics suffer from several shortcomings, which were encountered as the majority of bioplastics are unable to stand if used alone. The hydrophilic nature of seaweed, with its poor water barrier and antimicrobial properties, has limited its use for packaging applications [15]. To overcome these challenges, either organic or inorganic fillers that are less hydrophilic or hydrophobic are blended with raw seaweed or seaweed-derived polymers to broaden their applications [16]. The effectiveness of introducing organic fillers to improve the seaweed-based bioplastic film has been previously reported [17]. Hence, the use of organic fillers derived from solid biowaste is an excellent candidate for reinforcing materials in seaweed films due to their cost-effective, abundant, and eco-friendly nature.

Lignin is one of the cheapest and most abundant bioorganic polymers on earth. Lignin polymer, a by-product available in comparatively large numbers, has been extensively used for the production of new innovative bioplastics products [18]. Its valorization as reinforcement in bioplastics is a comparatively new and emerging research field. It has also been used as a filler due to its abundant, non-toxic, renewable, and excellent biodegradable properties [19]. This bioorganic polymer has hydroxyl functional groups employed in chemical reactions and allows its efficient valorization into value-added materials [20]. The precursor materials and processing procedure are used to influence the physicochemical characteristics of isolated lignin [21]. This renders differences in molecular weight, elemental composition, and functional groups and makes it interactable with many other polymers and shifts their functional properties. It has been acknowledged as a potential alternative to oil-derived materials due to its beneficial properties. In addition, its native role as a mechanical support in crops has contributed to its becoming a potential structural filler for biopolymer composites [22].

The present work is focused on the preparation and characterization of *Kappaphycus alvarezii* bioplastic films reinforced by LNPs. The LNPs were isolated from an empty fruit bunch of soda pulping waste. The isolation of lignin from industrial waste is a prime contribution to waste valorization, particularly in the pulp and paper industry. In this study, a novel approach has been proposed to enhance the properties of LNPs through a purification process to eliminate natural impurities such as lipids, waxes, fatty acids, and tannins. The present approach was compared with that of the unpurified LNPs towards the characteristics. These two types of LNPs were then used as reinforcing nanofillers in a *Kappaphycus alvarezii* matrix. Several techniques and analysis methods investigated the functional properties of the bioplastic film networks. This is the first report on the antimicrobial, water barrier, structural, and biodegradability characteristics of *Kappaphycus alvarezii* reinforced with unpurified LNPs or purified LNPs. The innovative bioplastic films were also compared with the unreinforced seaweed film to investigate the potential effects and synergisms. The biological test against gram-positive and gram-negative microbes shows that a synergic influence of both types of nanofillers in the matrix networks was achieved, indicating excellent antimicrobial activity against all two bacteria.

## 2. Materials and Method

### 2.1. Materials

Raw *Kappaphycus alvarezii* seaweed was purchased from Green Leaf Synergy Sdn. Bhd. (Tawau, Sabah, Malaysia). The Division of Bioresource Technology, Universiti Sains Malaysia (USM) supplied the black liquor of oil palm empty fruit bunches (EFB) from a soda pulping process. The practical grade of chemicals used in this study was purchased from Sigma-Aldrich (St. Louis, MI, USA). The chemicals used were of analytical grade: sulfuric acid (H_2_SO_4_), cyclohexane (C_6_H_12_), diethyl ether ((C_2_H_5_)_2_O), ethanol (C_2_H_5_OH), and glycerol (C_3_H_8_O_3_).

### 2.2. Isolation and Purification of Lignin

Lignin was isolated from the black liquor of EFB using the H_2_SO_4_ precipitation technique [23]. The liquor’s pH value was lowered using 4.84 M H_2_SO_4_ by slowly dripping it into the liquor until its pH reached 2.0. The precipitate was filtered using Whatman #42 in a *Büchner funnel* and washed with hot distilled water until the pH value reached neutral (~7). Thereafter, the slurry of lignin cake in the filter paper was dried overnight in an oven at 60 °C and tagged as unpurified lignin. The lignin was then purified with the process of chemical extractions. The lignin powder was stirred in (C_2_H_5_)_2_O to remove fats and fatty acids, followed by C_6_H_12_/C_2_H_5_OH (1:1, *v*/*v*) extraction in the soxhlet apparatus to remove lipids, tannins, and waxes [24]. The obtained lignin was washed for 30 min with warm distilled water (70 °C), dried at reduced pressure overnight at 85 °C, and recorded as purified lignin.

### 2.3. Preparation of LNPs

A high-energy ball milling was used to fabricate the nanostructure of unpurified and purified LNPs. It was operated at a rotational speed of 170 rpm for 24 h in an ambient environment. The stainless steel chamber (horizontal ball milling) was loaded with ball and lignin powder in a ratio of 10:1 (*w*/*w*). Three diameter sizes of stainless steel balls of 20 mm × 12 mm × 10 mm were used in this research. The LNPs were then finally dried overnight in an oven at 110 °C and stored in a zip lock bag inside a desiccator.

### 2.4. Characterization of LNPs

The morphological properties of LNPs were analyzed using transmission electron microscopy (TEM), energy-filtered EFTEM Libra 120-Carl Zeiss instrument (Oberkochen, Germany). Particle size measurement of LNPs was determined through dynamic light scattering (DLS) on a Malvern Zetasizer Nano ZS Ver. 7.11 (Malvern Instruments, Malvern, UK). The X-ray diffraction (XRD) analysis was carried out via a Bruker D8 Advance X-ray Diffractometer (Karlsruhe, Germany), and the sample data were collected at 2θ between 10° and 40°.

### 2.5. Fabrication of Lignin Nanoparticles/Kappaphycus Alvarezii Bioplastic Films

In this study, bioplastic films were prepared by the solution casting method. The steps were as follows: five grams of dried *Kappaphycus alvarezii*, as the base matrix, was dissolved in 250 mL distilled water and glycerol (50% *w*/*w Kappaphycus alvarezii*) in a beaker, where glycerol was used as a plasticizer. With reference to the dry weight of the red seaweed, unpurified and purified LNPs were added at different loading percentages of 0%, 1%, 3%, 5%, and 7%, respectively. The solution was heated to 90 °C for 60 min with continuous stirring and left to settle down to room temperature. The solution was then cast in a 20 cm × 20 cm square area tray and dried for 24 h in a ventilated oven at 40 °C and 50% relative humidity. Five replicates of each sample were cut out for further analysis.

### 2.6. Characterization of LNPs/Kappaphycus Alvarezii Bioplastic Films

#### 2.6.1. Morphological Analysis

Morphological analysis of the LNP filler’s loading compatibility with the *Kappaphycus alvarezii* matrix was observed under a scanning electron microscope. Surface morphologies of films were observed with FESEM (FEI Quanta FEG 650, Thermo Fisher Scientific, Eindhoven, The Netherlands). The surface roughness of the films was determined using atomic force microscopy (AFM) XE-70 Park System (Suwon, Republic of Korea).

#### 2.6.2. Structural Analysis

A Shimadzu’s Fourier transform infrared (FT-IR) spectroscopy, IR Prestige-21 (Kyoto, Japan), was used to analyze the specific surface organic functional group. The spectra were carried out from 4000 to 400 cm^−1^ at ambient temperature. The XRD analysis was carried out via a Bruker D8 Advance X-ray Diffractometer (Karlsruhe, Germany), and the sample data were collected at 2θ between 10° and 40°.

#### 2.6.3. Hydrophobicity and Physical Properties

The contact angle was determined with the sessile drop method on KSC CAM 101 (KSV Instruments Ltd., Espoo, Finland) at room temperature. Five measurements were recorded at different positions on the films to determine the average. The water vapor permeability (WVP) test was performed according to the ASTM E–96, 1996 method. The permeability cups filled with 30 mL of distilled water were mounted over the cups and sealed with the selected bioplastic films, and subjected for 6 h at 25 °C and 50% relative humidity (RH). The WVP (g·m·m^−2^·s^−1^·Pa^−1^ × 10^−10^) of the bioplastic films with the average of five samples was calculated using Equation (1) where WVTR was the measured water vapor transmission rate through a film, t was the mean film thickness (m), s was the saturation vapor pressure at a temperature 25 °C, R_1_ was the relative humidity at vapor source, and R_2_ was the relative humidity at vapor sink.

(1) $$\mathrm{WVP}=\frac{\mathrm{WVTR}\;\times \;t}{s\;\times \left(R_{1}-R_{2}\right)}$$

Water solubility (WS) was determined via the method described in a previous investigation [25]. Samples were cut to standard sizes (3 cm × 3 cm) and conditioned for three days in a desiccator with silica gel. The film specimens were then weighed, placed in 80 mL of distilled water, and agitated at 100 rpm at room temperature for 1 h. Thereafter, the film leftover was filtered with filter paper and oven-dried at 60 °C until a constant weight was achieved. Five replicates of each sample were measured, and the mean values were calculated. The solubility of the films was calculated according to Equation (2).

(2) $$\mathrm{WVP}\;(\%)=\frac{\mathrm{Initial}\;\mathrm{weight}-\mathrm{final}\;\mathrm{weight}}{\mathrm{Initial}\;\mathrm{weigt}}\;\times \;100$$

#### 2.6.4. Antimicrobial Properties

The antimicrobial activity was investigated with two food-borne pathogenic microbes: gram-positive microbes (*Staphylococcus aureus*) and gram-negative microbes (*Escherichia coli*) using the agar diffusion method. The bacteria of 10^7^ CFU (colony forming units)/mL were cultured in agar media with the sterile solution at 37 °C for 24 h. For the analysis, the sample films were punched into a 6 mm disk diameter. All the specimen films were subjected to a Petri dish and incubated for 24 h at 37 °C in an incubator. After 24 h, the inhibition zone of the bioplastic films was investigated using a caliper to measure the ability of the films to inhibit bacterial growth and was determined by mm as the diameter of the zone. The analyses were performed in five replicate specimens for each film.

#### 2.6.5. Soil Burial Test

Bioplastic film degradation was evaluated by weight loss during the soil burial test. All the film specimens with a size of 3 cm × 3 cm were weighed to determine the initial dry weight of the film before being buried beneath 5 cm of soil in an ambient environment with a moisture content of 36% (*w*/*w*). The soil medium was sprayed with water every 2 days to maintain the moisture and microorganism activity. The sample films were taken from the medium, carefully cleaned, and dried for 24 h at constant weight at 40 °C and placed inside a desiccator before weighing to obtain the dry weight of film residue at various time intervals within 10 days during 40 days of biodegradation. Five replicates of each film were weighed, and the mean values were calculated. The difference in weight loss of the sample was determined according to Equation (3), adapted from [7]:

(3) $$\mathrm{Weight}\;\mathrm{loss}\;(\%)=\frac{\mathrm{Initial}\;\mathrm{dry}\;\mathrm{weight}\;\mathrm{before}\;\mathrm{burial}\;-\;\mathrm{dry}\;\mathrm{weight}\;\mathrm{after}\;\mathrm{burial}}{\mathrm{Initial}\;\mathrm{dry}\;\mathrm{weigth}\;\mathrm{before}\;\mathrm{burial}}\;\times \;100$$

#### 2.6.6. Statistical Analysis

The data of hydrophobicity, physical, antimicrobial, and biodegradability properties were analyzed with statistical computation, one-way ANOVA using DSAASTAT ver.1.101 by Andrea Onofri. A post hoc multiple comparison test was performed by Tukey’s HSD to investigate the significant differences (*p* < 0.05) in the bioplastic films’ properties.

## 3. Results and Discussion

### 3.1. Characterization of Lignin Nanoparticles

**Figure 1:**
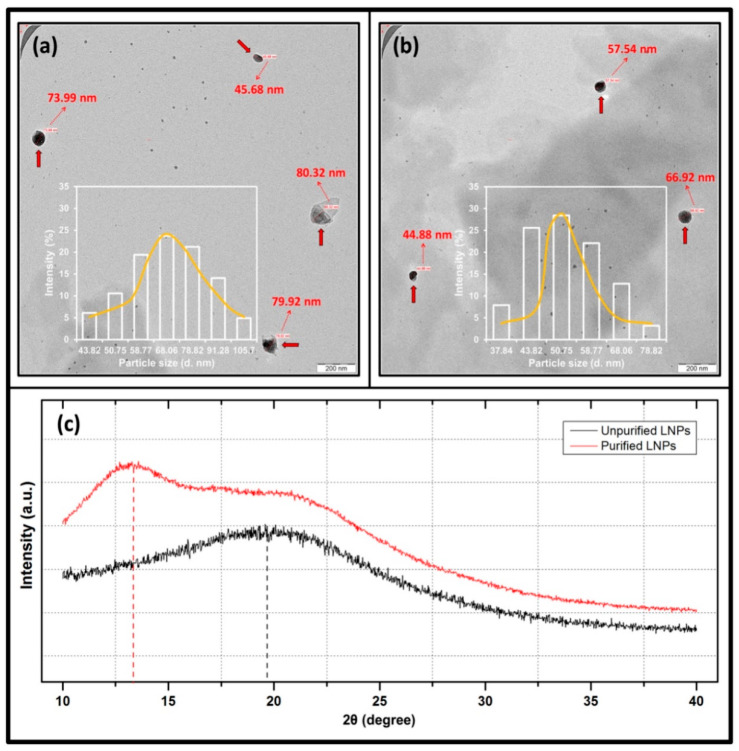
TEM micrograph and particle size distribution of (**a**) unpurified LNPs and (**b**) purified LNPs, and (**c**) XRD patterns of unpurified and purified LNPs.

The TEM micrograph and particle size distribution of unpurified and purified LNPs are displayed in Figure 1a,b. As seen in the figure, the unpurified and purified LNPs dispersed uniformly with particle sizes ranging from 46 nm to 80 nm and 45 nm to 67 nm, respectively. It is clear that all LNPs possessed an irregular shape and were free of the self-agglomeration of powder. Similar trends were also reported by a previous study that produced the LNPs from hardwood black liquor [26]. The shape of the LNPs evolved since the size of their structure was reduced (in particular, the purified LNPs) to the nanoscale during the ball milling process. The unpurified LNPs had a particle size distribution of 44–106 nm, with an average diameter of 70.34 nm. Meanwhile, the average diameter of purified LNPs was 52.84 nm, with a range of particle diameter of 38–79 nm. These findings were consistence with the result of TEM micrographs.

The particle diameter of LNPs presented a smaller diameter than the unpurified LNPs. This was probably due to the LNPs with the purification process had fewer extractive substances such as waxes, lipids, tannins, and fatty acids, which contributed to effective contiguity between the lignin particles and the ball mills during the ball milling process [27]. Enhanced functional properties of the nanobioplastic could be achieved, as the nanofiller is able to potentially charge the gaps and voids within the interfacial interaction [28]. This structure will act as a proper and sustainable nanomaterial to be employed as filler in bioplastic production.

Figure 1c depicts the X-ray powder diffraction analysis for both the unpurified and purified LNPs. The obtained diffractograms exhibited a wide area under the curve and showed no crystalline peaks, confirming the typical amorphous nature of both specimens. A single broad band centered at 19.59° was observed for unpurified LNPs. In previous investigations, a similar average peak at 20.26° was observed for kraft lignin [29], while alkali lignin appeared at an average peak at 22.8° [30]. In this study, however, the maximum 2θ value of LNPs was shifted to 13.58° after the purification process. This was attributable to the higher content of phenolic substances contained in purified LNPs than unpurified ones and the difference in chemical structures of the LNPs [31]. The deviation of 2θ value was probably caused by the purification protocol applied, which may change the composition of syringyl, guaiacyl, and *p*-hydroxyphenyl units where the contents of syringyl units were more than other units after purifying [32].

### 3.2. Characterization of Biopolymer Films Reinforced with Lignin Nanoparticles

#### 3.2.1. Morphological Analysis

**Figure 2:**
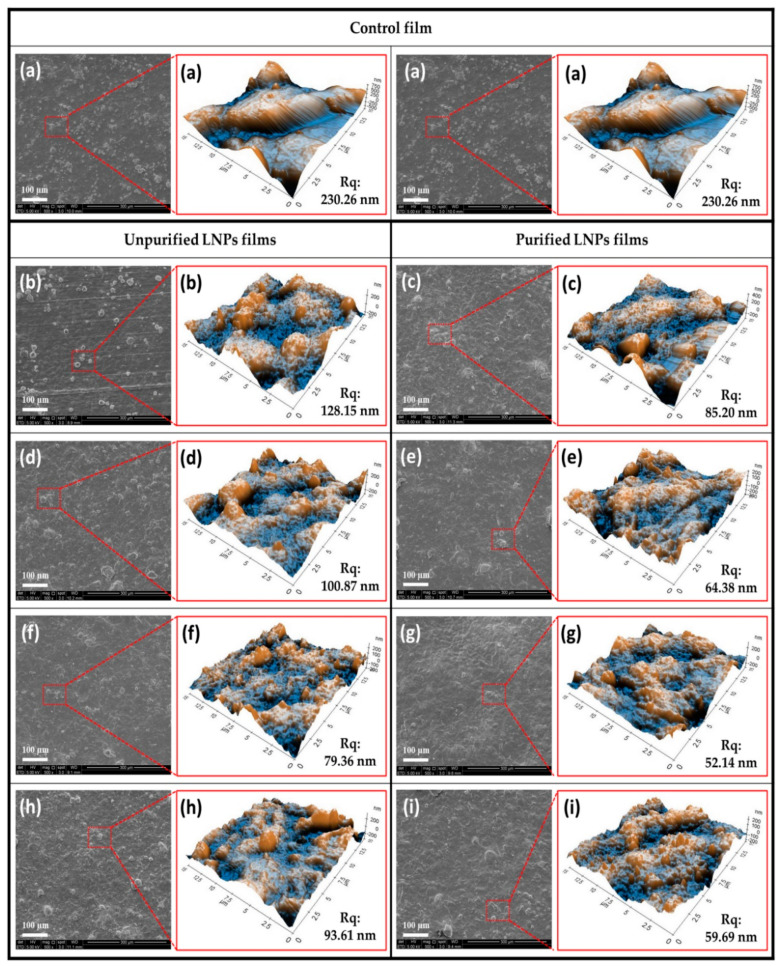
Surface morphologies by SEM and surface roughness by AFM of the bioplastic films for (**a**) control; unpurified and purified LNPs, (**b**,**c**) 1%, (**d**,**e**) 3%, (**f**,**g**) 5%, and (**h**,**i**) 7%.

The results of the surface morphology, topographical image, and the roughness coefficient of the unpurified and purified LNP-reinforced *Kappaphycus alvarezii* biopolymer films are presented in Figure 2. It can be observed from the figure that the control film showed coarse gaps on the surface. A similar tendency was found from the topographical image and roughness coefficient by the control film, both exhibit a high surface roughness value (Rq) of 230.26 nm. The incorporation of LNPs into biopolymer films improved the surface morphology and roughness. However, the surface micrographs of purified LNP films presented a smoother and homogeneous surface with a lower surface roughness compared to surfaces of control and unpurified LNP films. It was more noticeable with the composite film loaded with 5% purified LNPs. Homogeneity and well-dispersed nanofiller in the *Kappaphycus alvarezii* matrix were responsible for the lowest surface roughness of 52.14 nm [33].

In addition, more organized layers of purified LNP films were achieved compared to the films containing LNPs without purification protocol. This should be attributed to the absence of impurities in the purified LNP films [34]. A weaker interfacial interaction between the matrix and the unpurified nanofiller contributed to their poorer interfacial bonding, which is indicated by the presence of holes and uneven surfaces [35]. Meanwhile, beyond 5% incorporated nanofiller, a weak dispersion in the matrix networks was observed, which consequently contributed to increased surface roughness value and formation of voids at 7% of both LNPs loading. A similar finding was observed in a previous study, where the addition of nanofiller at high concentrations formed agglomerations due to poor dispersion, which diminished the surface roughness of the bioplastics [36].

#### 3.2.2. Structural Analysis

**Figure 3:**
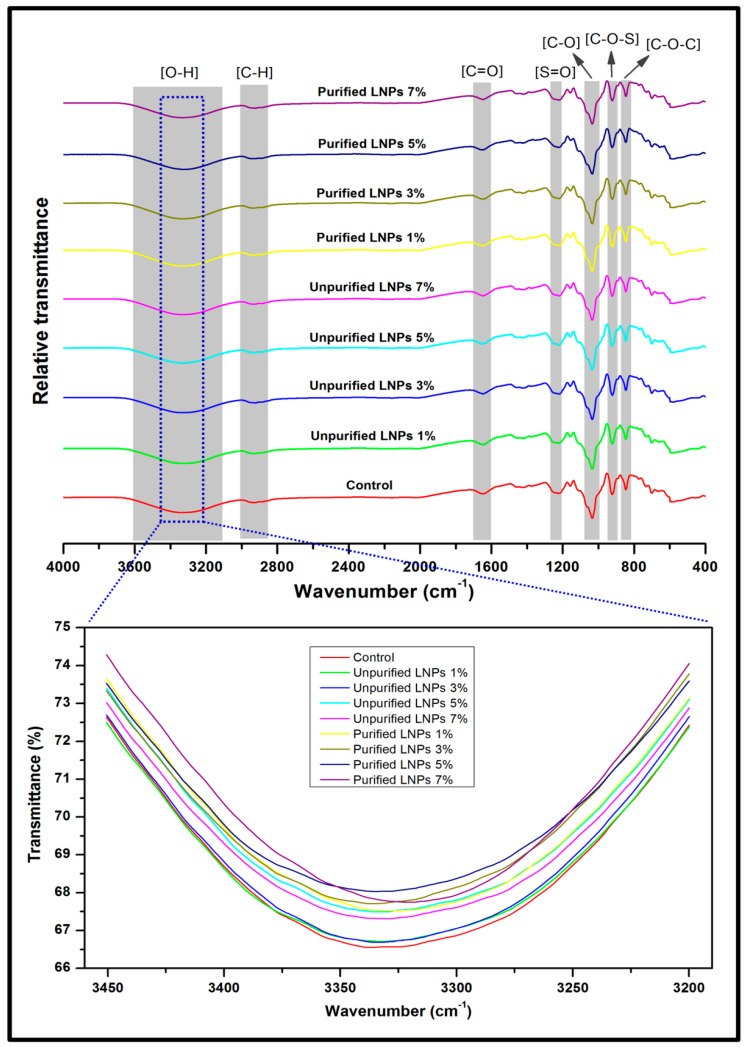
FT-IR spectra of control biopolymer film and bioplastic films reinforced by unpurified/purified LNPs.

Representative data on the surface chemical structure of the control and LNPs/*Kappaphycus alvarezii* biopolymer films are depicted in Figure 3. According to the FT-IR spectrum, the presence of O–H (hydroxyl) groups stretching the peak of all biopolymer film samples appeared in the broad region peak of 3100–3600 cm^−1^ [7]. The abundance of the interaction of free O–H groups in the pure film was associated with the hydrophilic nature of the red seaweed. The incorporation of unpurified and purified LNPs caused the absorption peak to shift to a lower wavenumber. However, the lower wavenumber of biopolymer films containing purified LNPs (especially at 5% loading) than neat film and unpurified LNPs films implies that the molecular interaction altered between the purified nanofiller and the seaweed matrix. As a result, the hydrophilicity of the bioplastic films was reduced due to the incorporation of LNPs. Similar findings were also reported by previous studies, where the use of lignin as filler in soy protein [37] and cassava starch [38] could contribute to a reduction of the bioplastic films’ hydrophilicity.

Furthermore, a vibration region of 2850–3000 cm^−1^ indicated the presence of the C–H (methyl) groups. A peak region of 1600–1700 cm^−1^ was associated with the appearance of C=O (carbonyl) groups bending, which is ascribed to the stretching vibration of carbonyl groups in the sulfated polysaccharides of *Kappaphycus alvarezii* [39]. The presence of an asymmetric stretching vibration of S=O (sulfate) groups of the kappa-carrageenan backbone appeared in the transmittance region of 1200–1250 cm^−1^. This functional group contributed to gelling characteristics of *Kappaphycus alvarezii* [40]. An assigned region band of 1000–1075 cm^−1^ indicated the C–O (alkoxy) groups from 3, 6-anhydro-D-galactose [41]. An intensity region band of 900–950 cm^−1^ for all bioplastic film samples was the indicator of C–O–S stretching in a (1-3)-D-galactose [42]. Meanwhile, the existence of C–O–C stretching in D-galactose-4-sulfate was identified in the region of 800–850 cm^−1^ [43].

**Figure 4:**
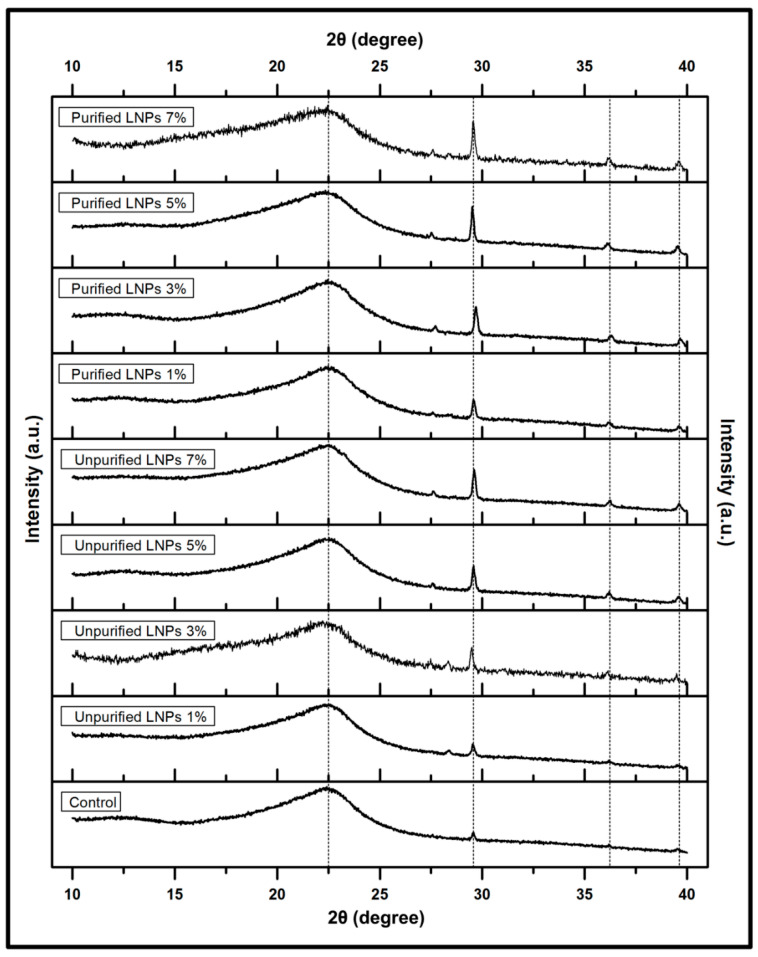
XRD patterns of control film and unpurified/purified LNP-reinforced bioplastic films.

Figure 4 shows the XRD measurement results of the control film and *Kappaphycus alvarezii* reinforced with unpurified and purified LNPs between 10° to 40° (2θ). According to the obtained diffraction patterns, all films achieved a broad peak between 20° to 25°, indicating the amorphous characteristics of the matrix. It can be observed that the neat film showed several other bands at 29.56°, 35.36°, and 39.51°, indicating the crystalline nature of *Kappaphycus alvarezii*. The crystalline band identified by XRD should be ascribed to the minerals (potassium chloride and sodium chloride) contained in the matrix. These findings were also consistent with previous works that reported similar phases of the red seaweed with the present work [44,45].

A very sharp band observed between 29° and 30° for all specimens of biopolymer films was probably due to the presence of calcite, a common mineral salt (calcite) formed naturally in *Kappaphycus alvarezii*. Apart from a considerable amount of carbohydrates, raw red seaweed contains various components of mineral salts such as magnesium, calcium, sodium, potassium, and iron [46]. As seen in the figure, the XRD profiles of the control film displayed lower intensity peaks than the biopolymer films incorporated with unpurified and purified LNPs. The addition of nanofillers into the seaweed matrix exhibited alteration of the diffractograms in position and intensity of the peak. The LNPs increased the diffraction peaks of the composite films as observed in the region peaks of 29°–30°, 35°–36°, and 39°–40°. According to the above analysis, this can be assumed that both LNPs were successfully incorporated into the *Kappaphycus alvarezii* matrix. Previous investigations also reported similar tendencies where the incorporation of lignin into biodegradable plastic films: polyvinyl alcohol [47], and polylactic acid [48], increased the diffraction peaks at similar regions.

#### 3.2.3. Hydrophobicity and Physical Properties

**Figure 5:**
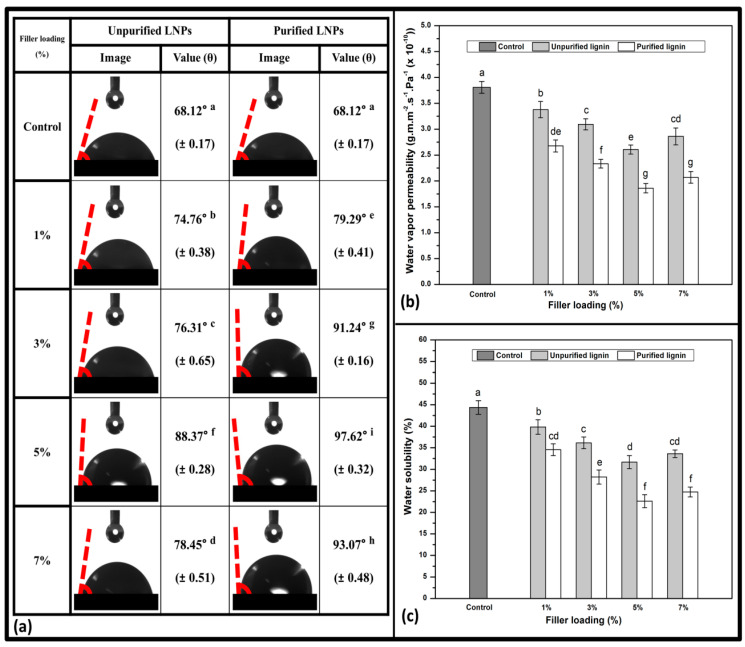
Hydrophobicity and physical properties of (**a**) contact angle, (**b**) water vapor permeability, and (**c**) water solubility of the LNP-reinforced bioplastic films. The same letters above the data bars indicate no significant difference in the values (*p* < 0.05).

The hydrophobicity and physical properties of the bioplastic films filled with LNPs are exhibited in Figure 5. The contact angle measurement was used to determine the surface hydrophobicity or wettability properties of the biopolymer films. As shown in Figure 5a, the control film had the lowest contact angle value of 68.12°. The addition of unpurified and purified LNPs noticeably improved (*p* < 0.05) the contact angle of the bioplastic films compared to the unreinforced film. This improvement might have occurred because of the strong intermolecular hydrogen bonding between the hydroxyl groups in the matrix and the LNPs [49]. This formation could generate a decrement in the number of free and available hydroxyl groups in the matrix to bind to the peripheral water molecules, resulting in a reduction in the affinity of the bioplastic film for water molecule permeability [50]. As a result, the hydrophobicity of the prepared bioplastic films was increased. Bioplastic film with 5% of purified LNPs loading provided the highest value of contact angle (97.62°). It reduced the water contiguity with the surface of the film and presented a solid droplet of water.

The purified films showed a superior contact angle than the unpurified ones. This denotes that the LNPs with purification enhanced film compatibility and miscibility within the *Kappaphycus alvarezii* matrix. Consequently, a reduction of porosity was observed, and more organized layers with lesser gaps in the bioplastic were achieved by introducing purified LNPs. However, a decreased trend of contact angle value loading was observed after achieving the optimum value. The LNPs at higher loading may probably have agglomerated in the bioplastic film [51]. This finding is in line with the results of the morphological properties of bioplastic films by SEM and AFM.

Water vapor permeability (WVP) is an essential parameter to measure the rate of moisture exchange between the films and the environment. Better moisture protection is normally indicated by a lower value of WVP [52]. Figure 5b depicts that all the reinforced bioplastic films possessed lower WVP values (*p* < 0.05) and showed significant differences compared to the *Kappaphycus alvarezii* itself. The bioplastic film with 5% purified LNPs provided the lowest WVP value of 1.86 × 10^−10^ g·m·m^−2^·s^−1^·Pa^−1^, while the film without incorporation of the nanofiller showed the highest WVP value of 3.81 × 10^−10^ g·m·m^−2^·s^−1^·Pa^−1^. The statistics rendered a noticeable difference in the nanofiller loading of unpurified and purified films, indicating that the addition of different loadings of LNPs, with or without purification, promoted an effect on the WVP behaviors of the prepared films. In addition, a significant difference in the WVP values between the two different groups of nanofillers was noticed. This should be attributable to the more uniform size and better miscibility of purified LNPs than unpurified LNPs. It appears that well dispersion, partial miscibility of the hydrophobic phenolic compounds of the LNPs, and the strong structure of hydrogen bonding with the matrix are able to fill up the voids. Hence, it formed more structural surfaces and smoother layers in the morphology. This characteristic induced a higher torturous pathway for the water molecule migration through the film as the LNPs were introduced, thereby leading to improved water barrier properties [53]. The obtained WVP values for the bioplastic films reinforced by LNPs fillers were better than the previously published report on biodegradable carboxymethyl cellulose films filled with organosolv lignins produced from corncob, which ranged from 2.93 × 10^−10^ g·m·m^−2^·s^−1^·Pa^−1^ to 3.61 × 10^−10^ g·m·m^−2^·s^−1^·Pa^−1^ [54].

**Figure 6:**
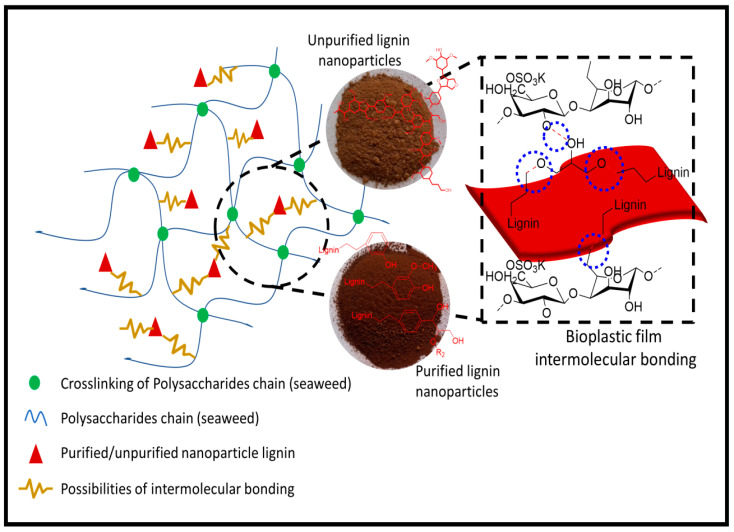
Proposed intermolecular bonding mechanism of *Kappaphycus alvarezii* bioplastic films functioned with LNPs.

The water solubility (WS) coefficients represent the resistance of the film formulation to water, which may be present on the material’s surface when the film is employed as a packaging medium and may also represent its intrinsic biodegradability [14]. As presented in Figure 5c, a pure polysaccharide is sensitive to water and moisture because of its inherent hydrophilicity. Blending LNPs with the red seaweed remarkably decreased the WS of the resulting films. This may be a result of the inclusion of the LNPs, rendering the film to be more invulnerably soluble in water. According to the obtained results, the WS value was significantly improved (*p* < 0.05), respective to the addition of the LNP fillers up to 5% into the biopolymer films. However, the incorporation of higher concentration of LNPs in *Kappaphycus alvarezii* led to a further increase of the WS values from 31.67% to 33.60% (unpurified LNPs) and from 22.61% to 24.73% (purified LNPs). The purified LNPs showed better improvement in the WS value than the unpurified and control film. This means enhanced hydrophobicity of the prepared films, exhibiting the purified LNPs, and the red seaweed reached a high degree of compatibility. It is one of the favorable behaviors for film functionality. The higher hydroxyl groups of phenolic compounds in purified LNPs formed a strong hydrogen bonding with the red seaweed matrix (Figure 6), resulting in improved compatibility in the matrix networks [55]. Similar behavior was also reported by previous work on chitosan films fabricated using lignin nanoparticles and acylated soy protein isolate nanogel [56]. In addition, the values of WS in this study were relatively greater than those previously reported [57], which used carrageenan as a matrix with the optimum WS value of 43.73%.

#### 3.2.4. Antimicrobial Properties

**Figure 7:**
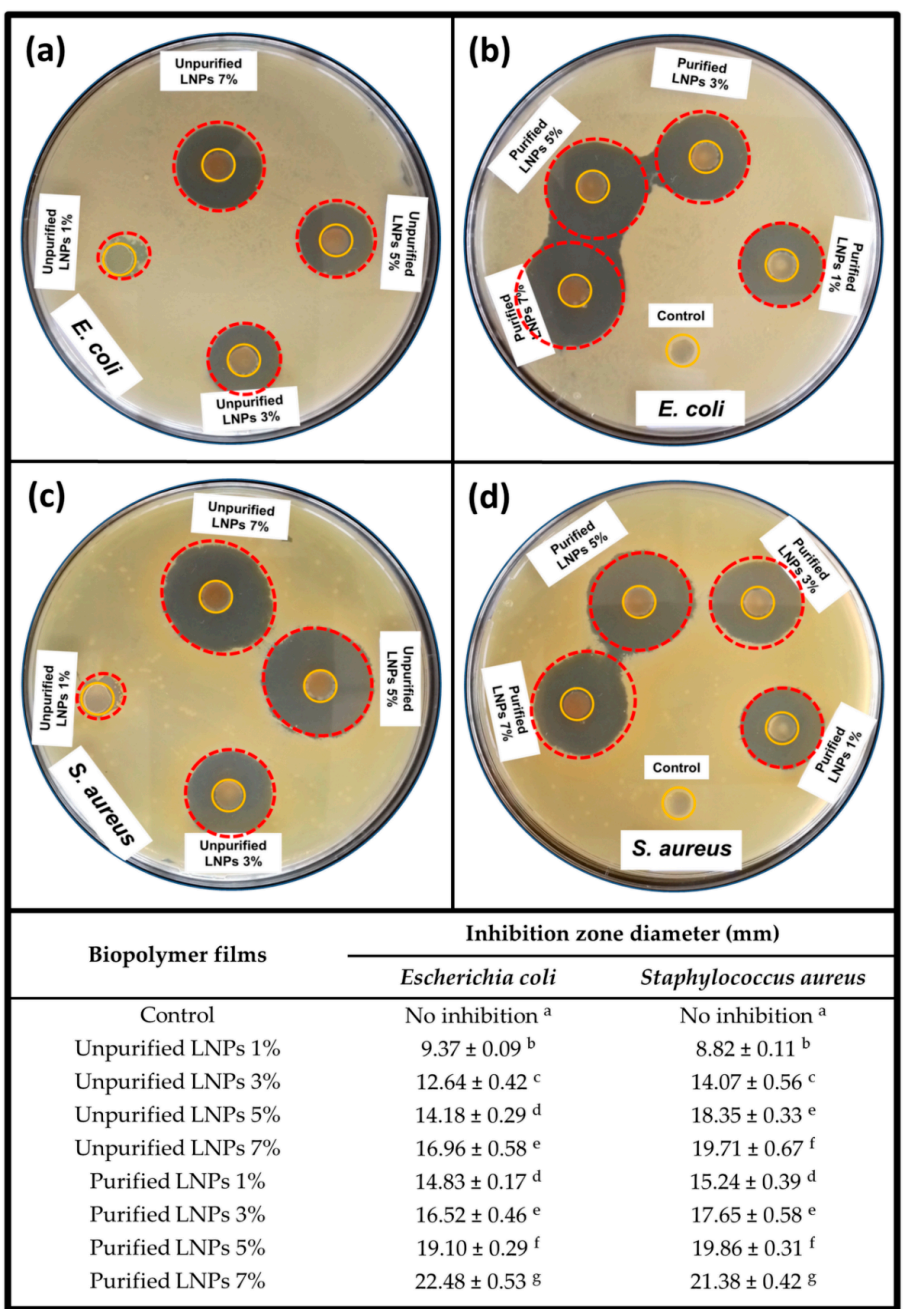
Photographic images and diameter statistics of inhibition zone measurement results of control film and unpurified/purified LNP films at different loadings against (**a**,**b**) *E. coli* and (**c**,**d**) *S. aureus*. Means of inhibition zone diameter followed by different superscript letters indicate significant differences (*p* < 0.05).

The antimicrobial of the *Kappaphycus alvarezii*/Lignin bioplastic film has not been explored yet. In this work, the antimicrobial properties of the bioplastic films were monitored against *Escherichia coli* and *Staphylococcus aureus*. These antimicrobial activities of the films were determined through the agar diffusion approach with the films in the disk plates, as indicated in Figure 7. The inhibition zone diameter surrounding the film disk shapes was applied to measure the antimicrobial activities and inhibitory zones of the bioplastic films. Interestingly, a previous study reported that lignin has excellent antimicrobial activity against food-borne pathogenic microorganisms [58]. The researchers found that bioactive compounds such as primarily alkylated phenols, hydroxycinnamic acid (p-coumaric and ferulic acids), syringol, and guaiacol-type in the lignin contributed to the antimicrobial properties of lignin since they are the fundamental antibiotics of the lignin utilized as a defense mechanism against different pathogens.

The pure *Kappaphycus alvarezii* film exhibited no appearance of an inhibition zone against the investigated bacteria. An inhibition zone was clearly observed in the films incorporated with LNPs. This difference was significant (*p* < 0.05) where the addition of 1% unpurified and purified LNPs into the films demonstrated an inhibition zone diameter of 9.37 mm and 14.83 mm for *Escherichia coli*, and 8.82 mm and 15.24 mm for *Staphylococcus aureus*, respectively. A noticeable progressive inhibition trend of the observed bacteria test specimens was recorded as the fraction of both LNPs was increased in the bioplastics structure. This indicates that the activity of anti-bacteria referred to the bioplastic film forming with nanosized lignin and the uniform dispersion in the *Kappaphycus alvarezii* matrix network [59]. In the case of unpurified LNPs, the optimum diameter of the inhibition zone against *Escherichia coli* and *Staphylococcus aureus* was obtained with 7% filler loading as 16.96 mm and 19.71 mm, respectively. With the same loading fraction, the optimum inhibitory zones of purified LNPs against *Escherichia coli* and *Staphylococcus aureus* were recorded as 22.48 and 21.38 mm, respectively. There was an obvious sterile zone surrounding the films, and both *Escherichia coli* and *Staphylococcus aureus* showed almost similar inhibitory zones at equal filler loading for each LNP and clear inhibitory effects. However, it is important to note that the purified films presented superior antibacterial activity compared to the unpurified films. This should be attributable to the higher amount of bioactive compounds in purified LNPs than LNPs without purification [60,61].

These findings signify that the antimicrobial properties of the bioplastic films were remarkably enhanced by the incorporation of LNPs in the matrix network, which provided significant effects on the ultimate properties of the bioplastic films. Interfacial interaction of the LNPs in the *Kappaphycus alvarezii* matrix and, subsequently, the effect of the purification process of the LNPs, improved the antimicrobial activity against *Escherichia coli* and *Staphylococcus aureus*. The obtained results were in good agreement with a previous study on polyvinyl alcohol/polyethyleneimine nanocomposite films with the incorporation of lignin as filler, where the antimicrobial activity (with the same type of food-borne pathogenic microorganisms) of the fabricated films was found to improve with the increase of lignin loading [62]. Similar tendencies to the present work were also previously reported in another investigation [63]. The authors found that the treatment via the demethylation process of lignin coatings on polyurethane films provided greater antibacterial properties compared to untreated lignin film.

#### 3.2.5. Biodegradability Studies

**Figure 8:**
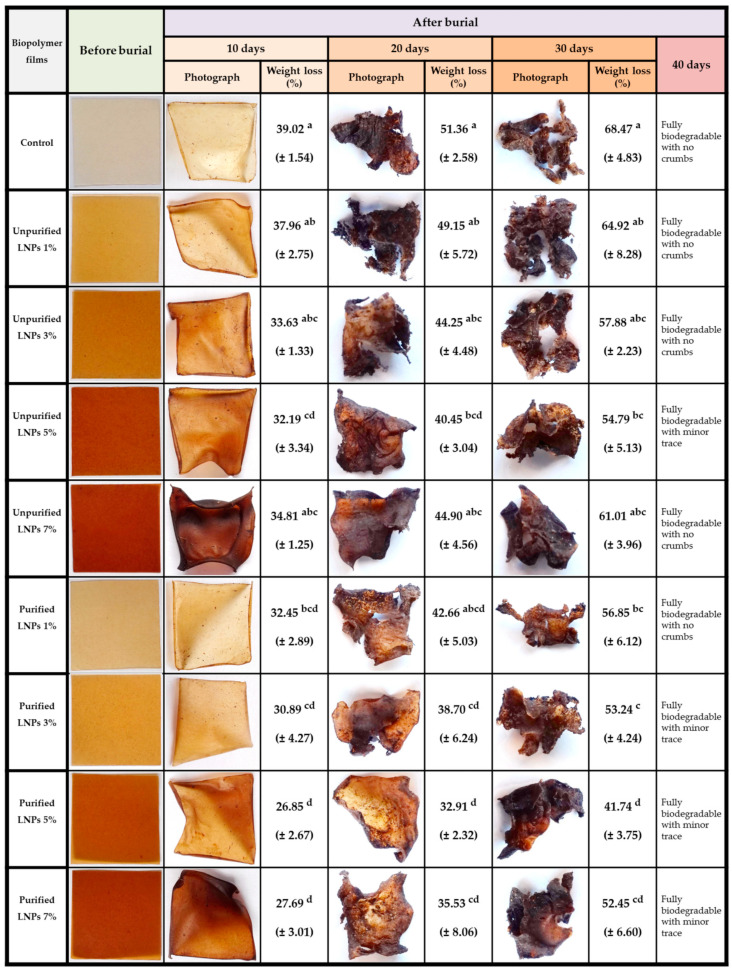
Digital images and weight loss before and after soil burial of biodegradable plastic films at 0, 10, 20, 30, and 40 days. Means in the same column followed by the same superscript letters denote no significant difference (*p* < 0.05).

The physical appearance and weight of degradation of the bioplastic films for a period of 40 days are displayed in Figure 8. It indicates that before the soil burial, all bioplastic films demonstrated relatively clear and smooth surfaces with a regular square appearance. A reduced trend of weight loss was achieved during degradation for all film specimens. The color change and degradation trends denote biological activity on the bioplastic films. Biodegradation took place after 10 days when all samples started to shrink and degrade. There was clearly a considerable increase in mass loss of degradation before burial to the tenth day. Although all samples tended to retain their original shape after the 10-day burial test, the obtained results showed that the pure *Kappaphycus alvarezii* film possessed the highest biodegradation rate of 39.02%, while the film with 5% purified LNPs exhibited the lowest weight loss of 26.85%. This was relative to the hydrophilic inherent of the red seaweed that could probably account for the rose biodegradation rate in the *Kappaphycus alvarezii* film itself.

The weight loss of the control sample was more prominent at 51.36% after the 20-day burial test, while the degradation rate of 40.45–49.15% and 32.91–42.66% were rendered by unpurified LP films and purified LNP films, respectively. The weight loss observed in LNP-reinforced red seaweed films was less prominent than that in the unreinforced films. This was probably due to the high phenolic compounds present in LNPs and the strong hydrogen bonding between LNP fillers and the matrix that also significantly improved the rigidity of the bioplastic films. After 30 days of soil burial, all the biodegradable films had undergone extreme shrinkage, turning cracks into crumbles with more weight loss. It was also observed in the figure that the color of all biodegradable films changed from yellowish to dark. These biodegradation findings are in agreement with those previously reported in another study on lignin/methylcellulose biocomposite films [64]. The researchers found that the biodegradation rate of lignin/methylcellulose films after being buried in soil for 30 days ranged from 46% to 69% with increasing lignin loading. Rapid degradation was found after 40 days of burial. Most of the bioplastic films exhibited no trace of cracking, and some showed a slight trace of crumbling.

Overall, it was found that the incorporation of LNPs into the *Kappaphycus alvarezii* film noticeably decreased (*p* < 0.05) the biodegradability of the films. Significant changes were also observed with the addition of different loadings and types of LNPs, where a small fraction of LNPs was introduced into the red seaweed matrix, and the biodegradability of films was decreased. Weight loss during biodegradation was reduced due to the decrement of microorganism attacks on the bioplastic films, as both LNPs (especially purified LNPs) could impede the attraction of microorganisms [65], which is not the case with the pure seaweed film. Naturally, both the nanofiller and the matrix are biodegradable polymers, and thereby the occurrence of microbial attack exists during the soil burial period. It appears feasible that the great properties and excellent biodegradability of LNPs/ *Kappaphycus alvarezii* blended films make them a high-potential candidate bioplastic material for sustainable packaging applications for dry stuff in the near future.

## 4. Conclusions

*Kappaphycus alvarezii* bioplastic films enhanced with unpurified LNPs and purified LNPs were successfully fabricated and characterized. The incorporation of LNPs noticeably enhanced the functional properties of the *Kappaphycus alvarezii* matrix structure compared to the control film. However, the purified LNP films were comparatively superior to unpurified LNPs in enhancing the surface roughness, hydrophobicity, water barrier, and antimicrobial properties. The optimum properties were presented by a bioplastic film with 5% purified LNPs, resulting in an Rq of 52.14 nm, a reduction in free available hydroxyl groups, a contact angle of 97.62°, a WVP of 1.86 × 10^−10^ g·m·m^−2^·s^−1^·Pa^−1^, a WS of 22.61%, and inhibitory zones against *Escherichia coli* of 22.48 mm and *Staphylococcus aureus* of 21.38 mm. All the obtained bioplastic films were fully biodegradable after 40 days of soil burial. Since the fabricated bioplastic films exhibited significant functional properties enhancement, they would be a sustainable long-term prospective candidate for replacing fossil-based plastics in packaging materials in an extensive range of applications.

## Data Availability

Not applicable.
